# Saffold Virus Type 3 (SAFV-3) Persists in HeLa Cells

**DOI:** 10.1371/journal.pone.0053194

**Published:** 2013-01-04

**Authors:** Toshiki Himeda, Takushi Hosomi, Takako Okuwa, Yasushi Muraki, Yoshiro Ohara

**Affiliations:** 1 Department of Microbiology, Kanazawa Medical University School of Medicine, Ishikawa, Japan; 2 The Public Health Institute of Kochi Prefecture, Kochi, Japan; Nagasaki University Graduate School of Biomedical Sciences, Japan

## Abstract

Saffold virus (SAFV) was identified as a human cardiovirus in 2007. Although several epidemiological studies have been reported, they have failed to provide a clear picture of the relationship between SAFV and human diseases. SAFV genotype 3 has been isolated from the cerebrospinal fluid specimen of patient with aseptic meningitis. This finding is of interest since Theiler’s murine encephalomyelitis virus (TMEV), which is the closely related virus, is known to cause a multiple sclerosis-like syndrome in mice. TMEV persistently infects in mouse macrophage cells *in vivo* and *in vitro*, and the viral persistence is essential in TMEV-induced demyelinating disease. The precise mechanism(s) of SAFV infection still remain unclear. In order to clarify the SAFV pathogenicity, in the present study, we studied the possibilities of the *in vitro* persistent infection of SAFV. The two distinct phenotypes of HeLa cells, HeLa-N and HeLa-R, were identified. In these cells, the type of SAFV-3 infection was clearly different. HeLa-N cells were lyticly infected with SAFV-3 and the host suitable for the efficient growth. On the other hand, HeLa-R cells were persistently infected with SAFV-3. In addition, the SAFV persistence in HeLa-R cells is independent of type I IFN response of host cells although the TMEV persistence in mouse macrophage cells depends on the response. Furthermore, it was suggested that SAFV persistence may be influenced by the expression of receptor(s) for SAFV infection on the host cells. The present findings on SAFV persistence will provide the important information to encourage the research of SAFV pathogenicity.

## Introduction

Saffold virus (SAFV) was identified from an infant with a fever of unknown origin in 2007 [1]. In the aid of phylogenetic analysis, SAFV was classified with Theiler-like rat virus, Theiler’s murine encephalomyelitis virus (TMEV) and Vilyuisk human encephalomyelitis virus into the species *Theilovirus* which belongs to the genus *Cardiovirus* of the family *Picornaviridae*. Eleven genotypes of SAFV have now been identified [1]–[6]. SAFV was isolated from nasal and stool specimens from infants presenting with respiratory or gastrointestinal symptoms. Furthermore, the virus was isolated from the cerebrospinal fluid (CSF) specimen of patient with aseptic meningitis. Although several epidemiological studies have been reported, they have failed to provide a clear picture of the relationship between SAFV and human diseases [7]. Recently, SAFV-2 was detected by RT-PCR in 2 children: in the CSF and a fecal sample from one child with monosymptomatic ataxia caused by cerebellitis; and in the CSF, blood, and myocardium of another child who died suddenly with no history of illness [8]. Moreover, animal experiments have been carried out and two different groups reported that SAFV is neurotropic in mice [9], [10]. These findings are of interest since TMEV, which is the closely related virus, is known to cause a multiple sclerosis-like syndrome in mice. TMEV persistently infects in macrophage cells *in vivo* and *in vitro*, and the viral persistence is essential in TMEV-induced demyelinating disease [11]. Therefore, the potential persistence of SAFV is an important issue. However, the precise mechanism(s) of SAFV infection still remain unclear.

It was reported that HeLa cells were most suitable cell line for the efficient growth of SAFV-3 [5]. However, we noticed that the growth of SAFV-3 in HeLa cells distinctly depends on the maintenance condition of the cells before infection. In the present study, the cell culture condition for the efficient growth of SAFV-3 and the potential persistence of SAFV-3 were studied by using two HeLa cell lines which are derived from two different laboratories.

## Materials and Methods

### Cells and Viruses

Two different HeLa cells were obtained from RIKEN (HeLa, RCB0007) and from Dr. Nishikawa (laboratory stock of Department of Biochemistry, Kanazawa Medical University School of Medicine) [12]. To avoid the confusion, the cells supplied from RIKEN were designated “HeLa-R” and the cells supplied from Dr. Nishikawa were designated “HeLa-N”. HeLa-R was maintained in Eagle’s minimum essential medium (MEM) (Nissui) supplemented with 10% newborn calf serum (CS) (Invitrogen) and 0.03% L-glutamine according to RIKEN’s recommendation. On the other hand, HeLa-N was maintained in Dulbecco’s modified Eagle’s medium (DMEM, SIGMA) supplemented with 0.03% L-glutamine and 10% fetal calf serum (FCS) containing 50 U/ml of penicillin and 50 µg/ml of streptomycin [12]. Additionally, in order to investigate the efficient maintenance condition for the virus growth, both cells were maintained for 2 weeks in several combinations of the above serum and medium. The detailed culture conditions for the corresponding cells are shown in Fig. S1. The appearance of cytopathic effect (CPE) was observed up to 48 hours post-infection (p.i.) and the CPE severity was graded from + to ++++ (+: <10% CPE, ++: 10∼50% CPE, +++: 50∼80% CPE, ++++: >80% CPE). BHK-21 cells were obtained from Cell Resource Centerfor Biomedical Research, Institute of Development, Aging and Cancer Tohoku University. BHK-21 cells were maintained in MEM supplemented with 5% CS and 0.03% L-glutamine.

The virus was prepared from infectious cDNA clone derived from JPN08-404 strain of SAFV-3, pSAF404 [13]. Briefly, pSAF404 were linearized with *Not* I, and RNA transcripts were synthesized with *CUGA* 7 RNA polymerase (Nippon gene). Then, HeLa-N cells were transfected with the transcripts derived from pSAF404 using Lipofectin (Invitrogen) according to the manufacturer’s instructions. The cultured cells and supernatants were collected after 48 hours, and the virus was prepared by three freezing/thawing cycles to release virions. Furthermore, the virus was propagated by two passages on HeLa-N cells. The virus titers were determined by a standard plaque assay on HeLa-N cells. The seed virus of DA strain of TMEV was propagated in BHK-21 cells. The culture cells and supernatants were collected after complete CPE was observed, and virus lysates were prepared by three freezing/thawing cycles to release virions. The virus titers were determined by a standard plaque assay on BHK-21 cells.

### Kinetics of Virus Growth in Cells

The kinetics of virus growth in HeLa-N and HeLa-R cells was analyzed. The cells were seeded at a density of 5×10^5^ cells in 35-mm dishes. After 24 h, the cells were infected with virus at a multiplicity of infection (MOI) of 10 plaque forming unit (pfu) per cell. After virus adsorption at 37°C for 60 min, the cells were washed twice with Dulbecco’s phosphate buffered saline (PBS), and incubated at 37°C in each medium with 1% serum. The cells and supernatants were collected at 0, 3, 6, 12, 24 and 48 h after infection and the viruses were prepared by three freezing/thawing cycles from the cells. SAFV-3 and DA viruses were titrated by a standard plaque assay on HeLa-N and BHK-21 cells, respectively.

### Analysis of Short Tandem Repeat (STR) for Identification of Two Different HeLa Cells

In order to investigate whether HeLa-N and HeLa-R cells are genomically identical, the STR on genome was analyzed [14]. Analysis of STR was outsourced by BEX co. ltd. (Tokyo, Japan) using Cell ID System (Promega).

### Neutralization Test

In order to generate an anti-SAFV-3 antibody for control, rabbits were immunized with SAFV-3 (JPN08-404) propagated in LLC-MK2 cells in TiterMax Gold (TiterMax USA) a few times at 1-week intervals, followed by two booster injections 1 month after the last immunization.

The titer of the challenge virus was determined on HeLa-N cells before the neutralization test was carried out. Two-fold dilutions of CS, FCS and anti-SAFV-3 antiserum were prepared by serum-free DMEM. Each sample serum (100 µl) was incubated with the challenge virus (100 TCID50/100 µl) at room temperature for 60 min. The virus-serum mixtures were inoculated into 96 well-plate containing HeLa-N cells. The cells were observed for CPE daily for 4–5 days.

### Establishment of HeLa-R Cells Persistently Infected with SAFV-3

HeLa-R cells (maintained with CS) and HeLa-N cells (maintained with FSC or CS) were seeded at a density of 5.0×10^6^ cells in a T75 flask. After 24 hours, the cells were infected with SAFV-3 at an MOI of 10 pfu per cell. After virus adsorption at 37°C for 60 min, the cells were washed twice with PBS, and incubated at 37°C in fresh medium with 1% serum. After 72 hours, surviving cells were washed twice by PBS and continuously cultured in fresh medium with 10% serum.

### Western Blotting

The HeLa-R, PSAF/HeLa-R (5 passages, 30 days p.i.) and HeLa-N infected with SAFV-3 (18 hours p.i.) cells were lysed in sodium dodecyl sulfate (SDS)-polyacrylamide gel electrophoresis (PAGE) sample buffer (10 mM Tris-HCl (pH8.0), 1 mM EDTA (pH8.0), 2.5% SDS, 5% 2-Mercaptoethanol, 10% glycerol and 0.005% bromophenol blue). Samples were separated by SDS-15% PAGE, and transferred onto a polyvinylidene difluoride membrane (Millipore). The membrane was blocked with 5% skim milk in PBS-T (PBS containing 0.05% Tween 20) for 60 min and incubated for 60 min with rabbit anti-SAFV-3 antiserum (1∶5,000) followed by incubation with horseradish peroxidase-conjugated anti-rabbit IgG (Bio-Rad Laboratories) for 60 min. Signals were detected using ECL plus Western blotting detection reagents (GE Healthcare) according to the manufacturer’s instructions.

### Determination of the Titer of the Virus Produced from PSAF/HeLa Cells

PSAF/HeLa-R cells (7 passages, 42 days p.i.) were seeded at a density of 1×10^6^ cells in 35-mm dishes. Next day, the cells were washed twice with PBS and then incubated in fresh MEM with 10% CS. After 24 hours, the supernatants or the mixtures of the supernatants and cells were harvested. The viruses were prepared by three freezing/thawing cycles. The titers were determined by a standard plaque assay on HeLa-N cells.

### Treatment with Anti IFN-α and IFN-β Antibody

PSAF/HeLa-R cells (9 passages, 50 days p.i.) were seeded at a density of 50% confluent in 35 mm dishes. Cells were incubated in MEM supplemented with 10% CS containing mouse monoclonal anti-human IFN-α antibody (1 µg/ml, PBL Biomedical Laboratories) or rabbit polyclonal anti-human IFN-β antibody (120 U/ml, PBL Biomedical Laboratories). The medium was changed every 2 days.

### Immunofluorescene Staining

In order to use the anti-SAFV-3 antiserum for immunocytochemistry, 500 µl of the anti-SAFV-3 antiserum was absorbed at 4°C for 24 hours by the homogenate of 1.2×10^8^ cells of HeLa-R cells. After centrifugation, the supernatant was used for the immunocytochemistry.

PSAF/HeLa-R cells (13 passages, 62 days p.i.) cultured with CS and PSAF/HeLa-R cells (9 passages, 62 days p.i.) cultured with FCS for 12 days were seeded onto cover glasses coated by poly-L-lysine. After 24 hours, cells were washed with PBS and then fixed in 10% formalin for 20 min at 4°C. After three washes with PBS, cells were permeabilized with 0.25% triton X-100 in PBS for 20 min at room temperature and blocked with 5% bovine serum albumin (BSA) in PBS for 60 min at room temperature. Cells were incubated with the anti-SAFV-3 antiserum in PBS supplemented with 5% BSA for 60 min at room temperature. After five washes with PBS, cells were incubated with Alexa Fluor 594-conjugated anti-rabbit IgG (Molecular Probes) for 60 min at room temperature. Photomicrographs were obtained at room temperature with a microscope equipped with a digital camera (Axiovision, Carl Zeiss).

### Virus Binding Assay

HeLa-N and HeLa-R cells were seeded onto cover glasses. After 24 hours, cells were washed with PBS and then fixed in 10% formalin for 20 min at 4°C. After three washes with PBS, cells were blocked with 5% skim milk in PBS-T (PBS containing 0.05% Tween 20) for 60 min at room temperature without the permeabilization. Cells were incubated with SAFV-3 at an MOI of 100 pfu per cell for 60 min at 4°C. After five washes with PBS-T, cells were incubated with the anti-SAFV-3 antiserum in PBS-T supplemented with 5% skim milk for 60 min at room temperature. After five washes with PBS-T, cells reacted with virus and antiserum were detected with Alexa Fluor 594-conjugated anti-rabbit IgG as described in the section of “Immunofluorescene staining”.

### Viral Titration after the Transfection of SAFV Recombinant Transcripts

HeLa-N and HeLa-R cells were seeded at a density of 5×10^5^ cells in 35-mm dishes. After 24 h, the cells were transfected with the SAFV recombinant transcripts (5 µg) derived from pSAF404 using DMRIE-C (Invitrogen) according to the manufacturer’s instructions. The cells and supernatants were collected at 16 h after transfection and the viruses were prepared by three freezing/thawing cycles from the cells. Viruses were titrated by a standard plaque assay on HeLa-N cells.

## Results

### Growth Kinetics of SAFV-3 on Two Different HeLa Cell Lines

**Figure 1:**
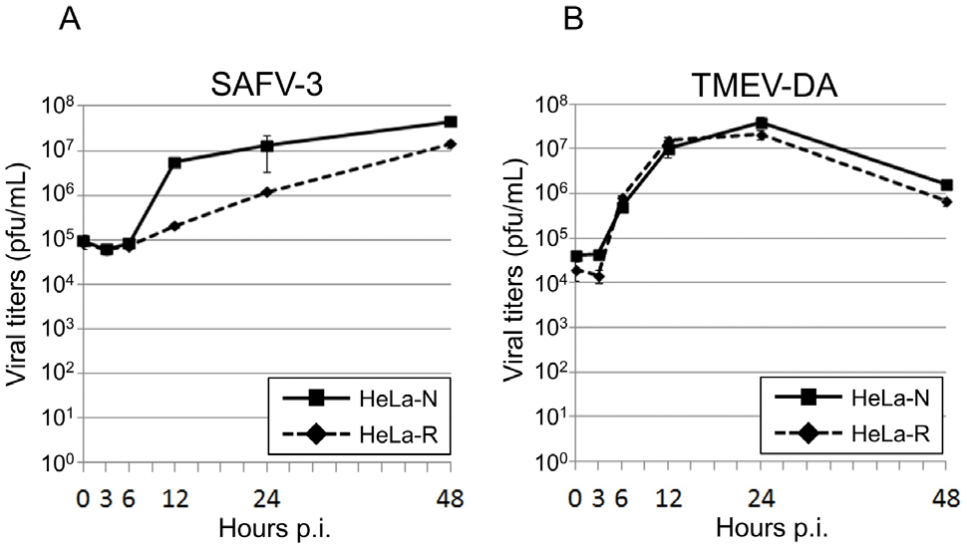
Growth kinetics of SAFV-3 and TMEV-DA on HeLa-N and HeLa-R cells. A: Growth kinetics of SAFV-3. Solid and broken lines indicate the growth curves of SAFV-3 on HeLa-N and HeLa-R cells, respectively. The viruses (as a mixture of cell-free and cell-associated viruses) were harvested at several time points indicated and assayed for titers by a standard plaque assay on HeLa-N cells. Titers shown are the means ± S.D. in three independent experiments. **B: Growth kinetics of TMEV-DA.** Solid and broken lines indicate the growth curves of TMEV-DA on HeLa-N and HeLa-R cells, respectively. The viruses (as a mixture of cell-free and cell-associated viruses) were harvested at several time points indicated and assayed for titers by a standard plaque assay on BHK-21 cells. Titers shown are the means ± S.D. in three independent experiments.

At first, the growth kinetics of SAFV-3 on two HeLa cell lines, which are derived from different laboratories, was analyzed. The titer of SAFV-3 produced from HeLa-N increased sharply at 12 hours p.i. (5.6×10^6^ pfu/ml) and gradually increased until 48 hours p.i. (4.4×10^7^ pfu/ml) (Fig. 1A, solid line). The virus produced from HeLa-R increased slowly at 12 hours p.i. (2.1×10^5^ pfu/ml) and gradually increased until 48 hours p.i. (1.4×10^7^ pfu/ml) (Fig. 1A, broken line). The virus growth on two cell lines was clearly different each other. At 12 hours p.i., the virus titer on HeLa-N was 1 log higher than that on HeLa-R. On the other hand, the titers of TMEV-DA on HeLa-N and HeLa-R cells were almost similar. Those peaked at 24 hours p.i. (4.0×10^7^ pfu/ml and 2.2×10^7^ pfu/ml, respectively) and decreased gradually (Fig. 1B). In addition, the plaques were not formed on HeLa-R cells by SAFV-3 infection (data not shown).

### Identification of Two Different HeLa Cell Lines by the STR Analysis

**Figure 2:**
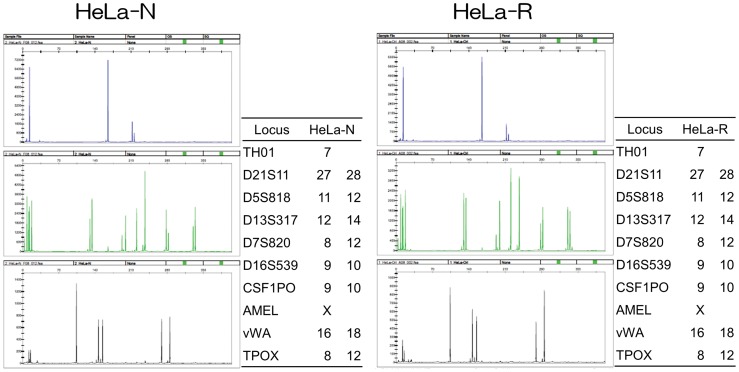
The STR analysis to confirm the identity of HeLa cells. Left and right panels show the data on HeLa-N and HeLa-R cells, respectively. Graphs were images generated by GeneMapper ver. 3.5 (Applied Biosystems). The allele data were presented as a table on the right side of each image. Locus indicates the name of gene analyzed. It is indicated that the gene presented by one datum was homo and the gene presented by two data was hetero.

The phenotypes of HeLa cells are varied in a number of laboratories. Furthermore, interspecies cross-contamination has been reported with HeLa cells [14]. Therefore, we performed the STR analysis to identify the two cell lines, HeLa-N and HeLa-R. Fig. 2 indicates that the STRs of HeLa-N and HeLa-R are identical, indicating that those are derived from the same origin, HeLa cell line (ATCC CCL2).

### Analysis of CPE on HeLa Cells Infected with SAFV-3 in Different Culture Conditions

Though it was demonstrated that HeLa-N and HeLa-R cells are identical genomically based on analysis of STR, the recommended maintenance conditions were different. Therefore, we next examined the effects of culture conditions on the growth of SAFV-3. HeLa-N and HeLa-R cells maintained with several conditions were infected with SAFV-3 at an MOI of 10 pfu per cell. The severity of CPE presented on each cell maintained with each condition was significantly different (Table.1). CPE on HeLa-N cells maintained in 10% FCS was the severest and that on HeLa-R cells maintained in 10% CS was the mildest. CPE on HeLa-N or HeLa-R cells maintained in 10% FCS was severer than that on HeLa-N or HeLa-R cells maintained in 10% CS. Interestingly, CPE on HeLa-N cells was severer than that on HeLa-R in the same conditions. No difference was observed between DMEM and MEM.

### Analysis of Anti-SAFV-3 Antibody and Other Inhibitors in CS

As shown in Table 1, whichever cells (HeLa-N or HeLa-R) are used, the virus growth (or the appearance of CPE) is suppressed in the culture of CS. Therefore, in order to investigate whether the antibody and/or the inhibitor(s) against SAFV are contained in CS, the neutralization test was performed. Even the 2-fold dilutions of FCS and CS did not inhibit the appearance of CPE on HeLa-N maintained with FCS which is the most susceptible to SAFV-3 infection, though the anti-SAFV-3 antiserum inhibits the appearance of CPE at 3,072-fold dilution. These results indicate that the antibody and/or inhibitor(s) against SAFV are not contained in CS.

**Table 1:** CPE presented by SAFV-3 infection on HeLa cells which were maintained in several culture conditions.

	HeLa-N				HeLa-R			
	FCS		CS		FCS		CS	
Hours p.i.	DMEM	MEM	DMEM	MEM	DMEM	MEM	DMEM	MEM
15 h	+++	+++	++	++	++	++	+	+
48 h	++++	++++	++++	++++	+++	+++	++	++

+: <10% CPE, ++: 10∼50% CPE, +++: 50∼80% CPE, ++++: >80% CPE.

### Establishment of HeLa-R Cells Persistently Infected with SAFV-3

**Figure 3:**
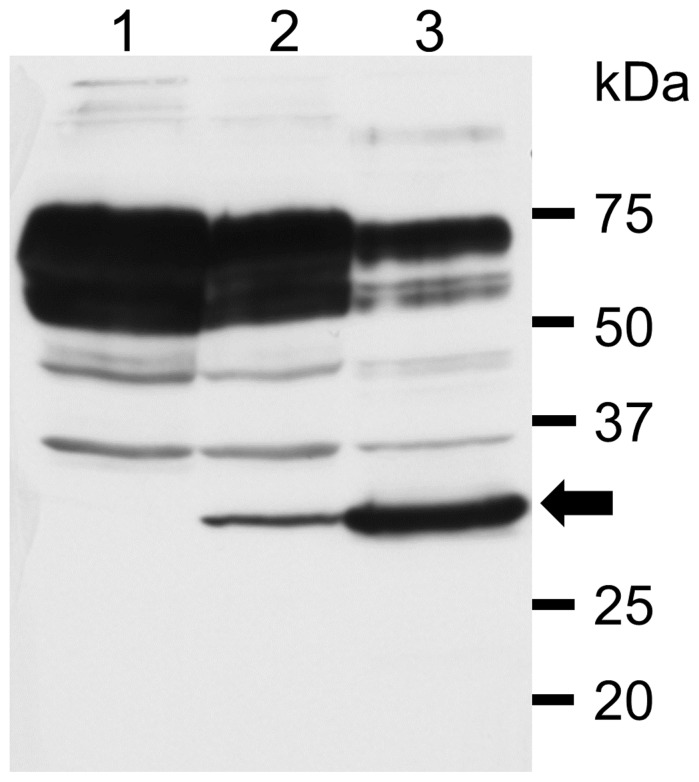
The detection of virus antigen in PSAF/HeLa-R cells by Western blotting. The anti-SAFV-3 antiserum detected the viral antigen of about 28∼30 kDa (arrow) in the lysates of PSAF/HeLa-R cells cultured for 30 days (5 passages) (lane 2) and HeLa-N cells infected with SAFV-3 (18 hours p.i.) used for a positive control (lane 3). The band of viral antigen was not detected in the lysate of HeLa-R cells used for a negative control (lane 1).

Since the CPE caused by SAFV-3 infection on HeLa-R cells maintained with CS was extremely mild, we attempted to establish the cells persistently infected with SAFV-3. At 72 hours p.i., surviving cells were harvested and sub-cultured in fresh MEM with 10% CS. The cells growing continuously were designated PSAF/HeLa-R cells. On the other hand, HeLa-N cells maintained with FCS or with CS did not survive. Even in the case of a low MOI of 0.1 pfu per cell, HeLa-N cells did not survive. In order to confirm the persistence of SAFV-3 on HeLa-R cells, Western blotting using the anti-SAFV-3 antiserum was performed. Viral antigen was detected in PSAF/HeLa-R cells cultured for 30 days (5 passages) (Fig. 3, lane 2). PSAF/HeLa-R cells cultured for 42 days (7 passages) produced infectious (cell-free) virus at 4×10^5^ pfu per 1×10^6^ cells within 24 hours after the medium was changed. The titer of cell-free virus from one PSAF/HeLa-R cell is calculated to be 0.4 pfu. On the other hand, total (cell-free and cell-associated) viruses were generated at 6×10^6^ pfu per 1×10^6^ cells within 24 hours. The titer of total viruses from one PSAF/HeLa-R cell is calculated to be 6 pfu. In addition, the direct sequencing of viruses produced from PSAF/HeLa-R cells demonstrated that the sequence of VP1 coding region is identical to that of SAFV-3 (JPN08-404). These data demonstrate that SAFV-3 persistently infects PSAF/HeLa-R cells *in vitro*. The persistent infection of SAFV-3 in PSAF/HeLa-R was maintained for 70 days (16 passages) at least. In these culture periods, clear CPE was observed in only a part of cells. Furthermore, the cell line which is resistant to SAFV infection was not established.

### Effects of Anti-IFN-α Antibody, Anti-IFN-β Antibody and FCS on PSAF/HeLa-R Cells

**Figure 4:**
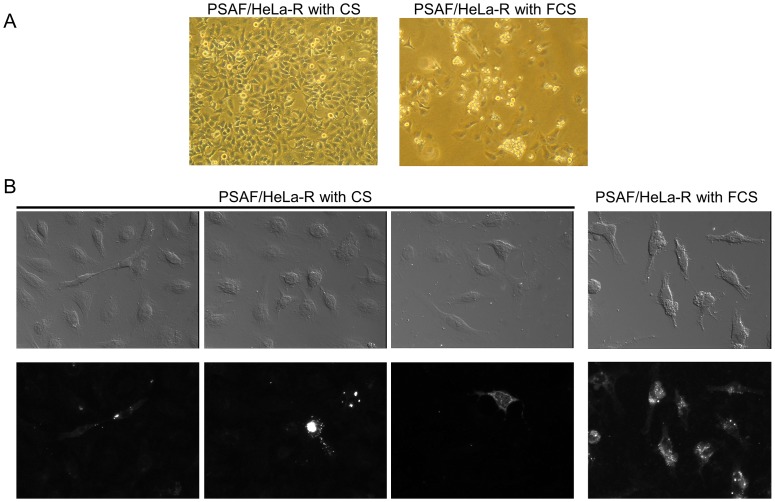
Effects of culture with FCS on PSAF/HeLa-R cells. **A: Representative microphotographs of the CPE on PSAF/HeLa-R cells** Left panel shows the PSAF/HeLa-R cells maintained in MEM with 10% CS (62 days p.i.). Right panel shows the PSAF/HeLa-R cells maintained in DMEM with 10% FCS for 12 days (from 50 days to 62 days p.i.). CPE on the PSAF/HeLa-R cells significantly increased by the culture with FCS for 12 days. Magnification: ×100. **B: Representative microphotographs of the immunocytochemistry** Left 6 panels show the PSAF/HeLa-R cells maintained in MEM with 10% CS. Right 2 panels show the PSAF/HeLa-R cells maintained in DMEM with 10% FCS. Upper and lower panels show Nomarski and fluorescent images, respectively. Virus antigen was detected with anti-SAFV-3 antiserum pre-absorbed by the homogenates of HeLa-R cells and Alexa Fluor 594-conjugated anti-rabbit IgG antibody. Viral antigen positive cells were shown in a part of PSAF/HeLa-R cells cultured with CS. After cultivation with FCS for 12 days, however, viral antigen positive cells clearly increased. Magnification: x400.

Although PSAF/HeLa-R cells (9 passages, 50 days p.i.) were treated by anti-IFN-α antibody (1 µg/ml/48 h) or anti-IFN-β antibody (120 U/ml/48 h) in order to investigate the involvement of IFN in the persistent infection, CPE on PSAF/HeLa-R cells did not increase even at 14 days post-treatment (data not shown). On the other hand, CPE on PSAF/HeLa-R cells clearly increased within 12 days by the change of the serum for cell maintenance from CS to FCS (Fig. 4A). Furthermore, virus antigen was detected in most of PSAF/HeLa-R cells cultured with FCS, although it was detected in a part of PSAF/HeLa-R cells cultured with CS (Fig. 4B). However, PSAF/HeLa-R cells maintained with FCS did not die out for the additional 2 weeks.

### Virus Binding Assay

**Figure 5:**
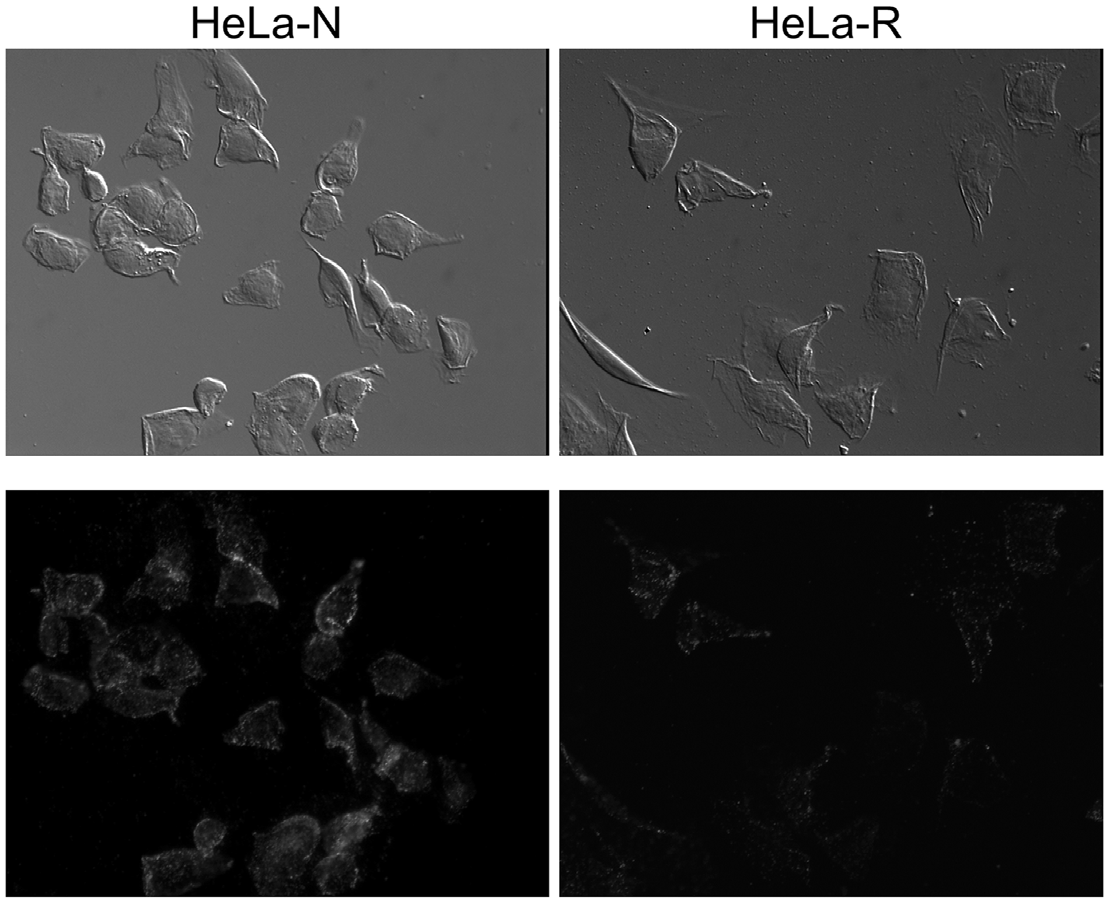
Immunofluorescent detection of the virus binding. The photos show the virus binding to the cell surface molecule(s) of each cell line. The cells fixed by 10% formalin were incubated with virus (MOI of 100). Then the viruses binding to the cell surface molecule(s) were detected by anti-SAFV-3 antiserum pre-absorbed by the homogenates of HeLa-R cells and Alexa Fluor 594-conjugated anti-rabbit IgG antibody. Left panels: HeLa-N cells, Right panels: HeLa-R cells. Upper and lower panels show Nomarski and fluorescent images, respectively. The viruses binding to cell surface of HeLa-R cells was significantly few, suggesting that the expression of receptor for SAFV infection is low in HeLa-R cells. Magnification: ×400.

In order to clarify whether SAFV persistence is dependent on the receptor expression, the virus binding assay was performed (Fig. 5). The viruses bound to the cell surface molecule(s) of HeLa-N cells were significantly numerous compared with those of HeLa-R cells.

### Viral Titration After the Transfection of SAFV Recombinant Transcripts

To obtain further evidence that receptor densities are the (main) cause of cell-type differences, viral titers were determined after transfection of SAFV recombinant transcripts in both cell lines (Table 2). The titers of cell-free and cell-associated SAFV produced from HeLa-N cells at 16 hours post-transfection were 9.3×10^1^ pfu/ml and 9.1×10^2^ pfu/ml, respectively. On the other hand, the titers of cell-free and cell-associated SAFV produced from HeLa-R cells at 16 hours post-transfection were 3.7×10^2^ pfu/ml and 1.1×10^3^ pfu/ml, respectively. The titers of SAFV produced from both cell lines after RNA transfection were equivalent level.

**Table 2:** The titers of viruses produced from HeLa-N and HeLa-R transfected with viral recombinant transcripts.

		Viral titers (pfu/mL)	
		Means	S.D.
HeLa-N	Sup	93.3	58.0
	Cell	913.3	96.1
HeLa-R	Sup	373.3	89.6
	Cell	1106.7	436.6

Viruses were harvested at 16 hours post-transfection. Titers shown are the means and standard deviations (S.D.) in three independent experiments.

## Discussion

SAFV was identified as a novel human cardiovirus in 2007 [1] although cardioviruses have been thought to mainly infect rodents. Several reports suggest that the virus may have diverse potential pathogenicity (e.g. respiratory illness, gastrointestinal illness, neurological diseases and/or type I diabetes) [7]. However, it is not clear that SAFV is the pathogen causing those clinical presentations. Since the potential persistence of SAFV is an important issue as described in “Introduction”, in the present study, the possibilities of the persistent infection of SAFV were analyzed.

We first investigated on the differences of the growth of SAFV-3 in two HeLa cell lines, HeLa-N and HeLa-R, which are derived from different laboratories. As shown in Fig. 1A, the growth kinetics of SAFV-3 was significantly different in these two subtypes of cells although the growth kinetics of DA strain of TMEV was similar in both cell lines (Fig. 1B). STR analysis demonstrated that HeLa-N and HeLa-R cells were genomically identical (Fig. 2). Therefore, it was thought that the maintenance in a different condition has changed the phenotype(s) of these cells, which affect the SAFV growth but not affect the TMEV growth. We next observed the CPE after SAFV infection on HeLa-N and HeLa-R cultured in several conditions (Table 1). CPE by SAFV-3 infection on HeLa-N cells was severer than that on HeLa-R cells in the same conditions. Furthermore, the CPE by SAFV-3 infection on HeLa-N cells cultured with FCS are severer than that on HeLa-N cells pre-cultured with CS for 2 weeks. However, HeLa-N cells incubated with 1% CS after infection (without 2-week pre-culture with 10% CS) showed the CPE almost similar to that observed in HeLa-N cells incubated with 1% FCS after infection (data not shown). In addition, the neutralization test demonstrated that CS does not inhibit the virus infection. These data suggest that the inhibitor(s) against SAFV-3 were not contained in CS. On the other hand, HeLa-R cells incubated with 1% FCS after infection (without pre-culture with 10% FCS) also showed the CPE almost similar to that observed in HeLa-R cells incubated with 1% CS after infection (data not shown). In addition, even 2-week pre-culture with FCS cannot induce CPE in HeLa-R cells as severely as in HeLa-N cells, suggesting that the appearance of CPE may not be due to a direct enhancement of virus growth by the contents of FCS. Therefore, the severity of CPE is thought to depend on the unknown host factors(s) induced by the long-period culture with FCS.

Milder CPE of HeLa-R cells cultured with CS led us to attempt to establish the persistent infection of SAFV-3 on HeLa-R cells cultured with CS. Surviving cells after infection could be easily sub-cultured in fresh MEM with 10% CS although HeLa-N cells died out. Survived sub-cultured cells were designated “PSAF/HeLa-R”. Persistent infection of SAFV-3 in PSAF/HeLa-R was demonstrated by Western blotting (Fig. 3) and plaque assay. The type of viral persistence of PDAJ774 cells, which is a murine macrophage-like cell line J774 persistently infected with TMEV-DA [15], belongs to the chronic focal infection according to the classification by Boldogh et al. [16]. In the case of PDAJ774 cells, the chronic focal infection was collapsed by the treatment with anti-mouse IFN-β antibody (80 U/ml/48 h) within 8 days [15], suggesting that it depends on type I IFN response of the host cells. However, the condition of PSAF/HeLa-R cells was not changed by the treatment with anti-human IFN-α antibody (1 µg/ml/48 h) or anti-human IFN-β antibody (120 U/ml/48 h) even at 14 days post-treatment. In addition, the number of virus antigen positive cells clearly increased in the culture with FCS for 12 days (Fig. 4B). These results suggest that the persistent infection of SAFV-3 in PSAF/HeLa-R cells does not depend on type I IFN response of host cells unlike the case of PDAJ774. In coxsackievirus, viral persistence is influenced by the cell cycle status [17] and/or the receptor expression [18]. Since the growth of HeLa-N and HeLa-R cells is similarly well in the medium supplemented with FCS or CS, the effects of the culture with FCS or CS on the cell cycle status is hardly analyzed. Therefore, we tried to compare the receptor expression of HeLa-N and HeLa-R cells by the virus binding assay, although the receptor for SAFV infection is not identified. Interestingly, the virus binding assay demonstrated that the expression of the virus binding molecule(s) (apparently SAFV receptor) on cell surface is significantly higher in HeLa-N cells cultured with FCS (Fig. 5). Additionally, to confirm the influence of the expression of receptor(s) to SAFV-3, cells were infected with SAFV-3 at a low MOI (0.5 pfu per cell) (Fig. S2). The viral antigen positive cells were significantly fewer in HeLa-R cells, suggesting that the efficiency of SAFV-3 infection in HeLa-R cells is low due to the low level expression of SAFV receptor(s). To further clarify this issue, viral titers after the transfection of the SAFV recombinant transcripts were determined in HeLa-N and HeLa-R cells. The results demonstrated that the (main) cause of cell-type differences is an entry suppression (apparently due to receptor densities), not a translation or replication block, since the both cell lines gave equivalent titers. These data suggested that SAFV persistence may depend on the receptor densities like the case of coxsackievirus [18]. The identification of the receptor(s) for SAFV infection will provide more critical evidence.

In conclusion, the present study demonstrated that SAFV is able to persist in human-derived cell line, HeLa cells. It brings up the possibility that SAFV may cause a persistent infection in humans as well as TMEV (the related virus) persists in mice. Furthermore, it was suggested that SAFV persistence may be influenced by the receptor expression of the host cells. The present findings of the *in vitro* SAFV-3 persistence will be helpful for further studies on the SAFV pathogenicity.

## Supporting Information

Figure S1 Diagram showing the culture conditions for HeLa-N or HeLa-R cells. The closed and opened triangles represent the time point of the change of serum and the virus infection, respectively.
(TIF)

Figure S2 Immunofluorescent staining of the cells infected with SAFV-3 at a low MOI. Left panels show HeLa-N cells at 24 hours p.i. of SAFV-3 (MOI of 0.5), Right panels show HeLa-R cells at 36 hours p.i. of SAFV-3 (MOI of 0.5). Upper and lower panels show Nomarski and fluorescent images, respectively. Viral antigen was detected by anti-SAFV-3 antiserum pre-absorbed by the homogenates of HeLa-R cells and Alexa Fluor 594-conjugated anti-rabbit IgG antibody. Magnification: ×400.
(TIF)
