# Correction: Saffold Virus Type 3 (SAFV-3) Persists in HeLa Cells

**DOI:** 10.1371/annotation/33ee31ba-3163-4859-ac37-7300ea78537e

**Published:** 2013-09-09

**Authors:** Toshiki Himeda, Takushi Hosomi, Takako Okuwa, Yasushi Muraki, Yoshiro Ohara

A grant from Kanazawa Medical University was incorrectly omitted in the Funding Statement. The first sentence of the Funding Statement should read: "This work was supported by a Grant-in-Aid for Young Scientists (B) from Japan Society for the Promotion of Science (24790449), Grant-in-Aid for Scientific Research (C) from the Ministry of Education, Culture, Sports, Science and Technology (22590421), a Grant for Promoted Research (S2012-6) and Collaborative Research (S2012-3) from Kanazawa Medical University, a Grant from Takeda Science Foundation, a Grant from the Neuroimmunological Research Committee of the Ministry of Health, Labor and Welfare, a Grant-in-Aid for Research on Emerging and Re-emerging Infectious Diseases and a Grant-in-Aid for the Promotion of Polio Eradication from the Ministry of Health, Labour and Welfare, Japan."

In addition, there was an error in the eighth sentence of the second paragraph of the "Cells and Viruses" section of Materials and Methods. The correct sentence is: "The culture cells and supernatants were collected after complete CPE was observed, and virus was prepared by three freezing/thawing cycles to release virions." 

