# Supplementary figures and images for: Saffold Virus Type 3 (SAFV-3) Persists in HeLa Cells
Source: PLoS One. 2013 Jan 4;8(1):e53194. doi: 10.1371/journal.pone.0053194 (PMC3537732; doi:10.1371/journal.pone.0053194)

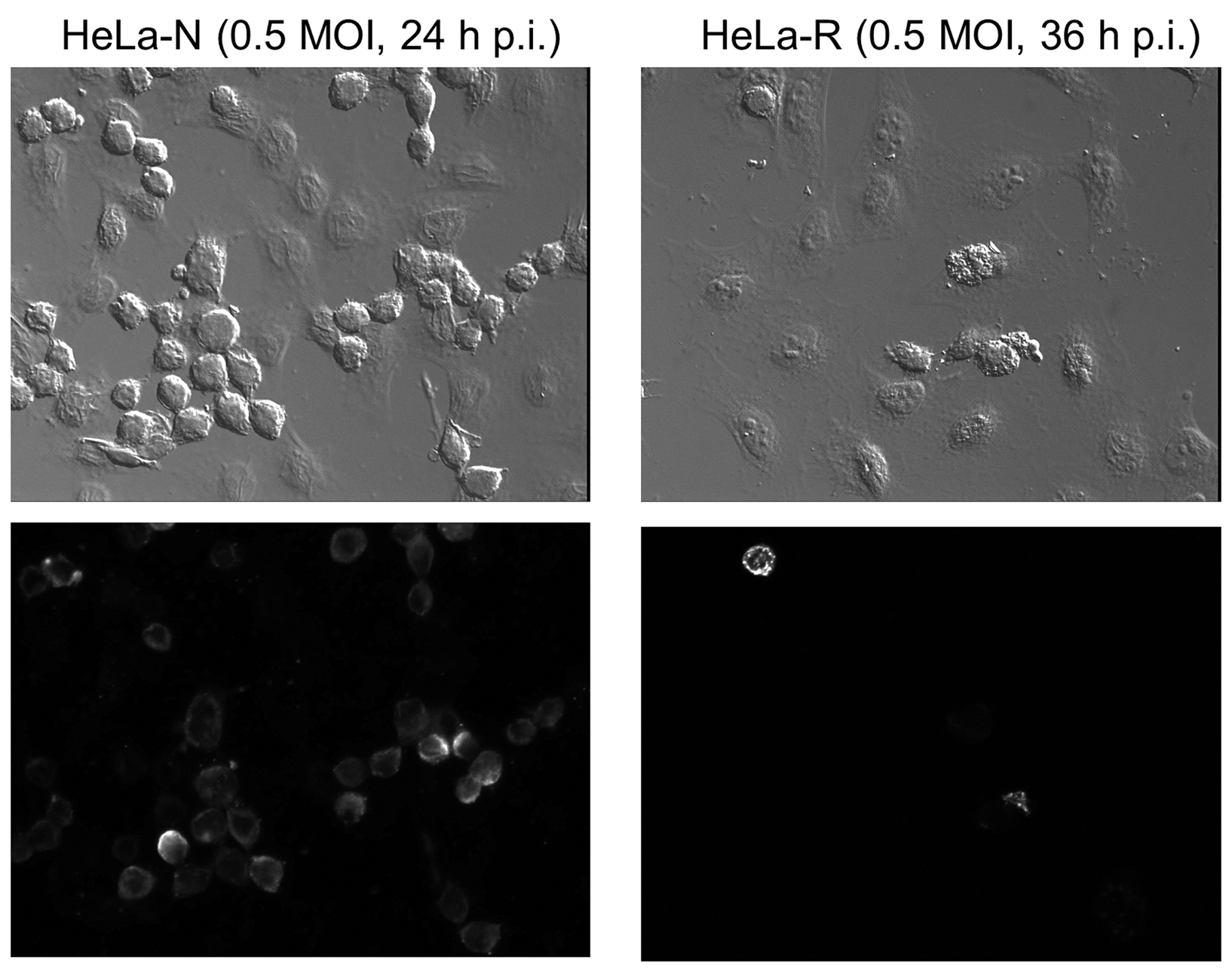

Supplement: Figure S2 — Immunofluorescent staining of the cells infected with SAFV-3 at a low MOI. Left panels show HeLa-N cells at 24 hours p.i. of SAFV-3 (MOI of 0.5), Right panels show HeLa-R cells at 36 hours p.i. of SAFV-3 (MOI of 0.5). Upper and lower panels show Nomarski and fluorescent images, respectively. Viral antigen was detected by anti-SAFV-3 antiserum pre-absorbed by the homogenates of HeLa-R cells and Alexa Fluor 594-conjugated anti-rabbit IgG antibody. Magnification: ×400. (TIF) [file pone.0053194.s002.tif]
